# Modelling the nicotine pharmacokinetic profile for e-cigarettes using real time monitoring of consumers’ physiological measurements and mouth level exposure

**DOI:** 10.1186/s13040-024-00375-z

**Published:** 2024-07-17

**Authors:** Krishna Prasad, Allen Griffiths, Kavya Agrawal, Michael McEwan, Flavio Macci, Marco Ghisoni, Matthew Stopher, Matthew Napleton, Joel Strickland, David Keating, Thomas Whitehead, Gareth Conduit, Stacey Murray, Lauren Edward

**Affiliations:** 1 B.A.T. (Investments) Limited, Regents Park Road, Millbrook, Southampton, SO15 8TL UK; 2 Hidalgo LTD, Unit F Trinity Court Buckingway Business Park, Anderson Road, Cambridge, CB24 4UQ UK; 3 Zizo, 8 Douglas House, Bletchley, MK1 1BA UK; 4 Intellegens, The Studio, Chesterton Mill, Cambridge, CB4 3NP UK; 5 B-Secur LTD, Catalyst Inc, The Innovation Centre, Queen’s Road, Belfast, BT3 9DT UK

**Keywords:** Pharmacokinetic, Physiological Measurements, Plasma nicotine, Heart Rate, Machine Learning

## Abstract

Pharmacokinetic (PK) studies can provide essential information on abuse liability of nicotine and tobacco products but are intrusive and must be conducted in a clinical environment. The objective of the study was to explore whether changes in plasma nicotine levels following use of an e-cigarette can be predicted from real time monitoring of physiological parameters and mouth level exposure (MLE) to nicotine before, during, and after e-cigarette vaping, using wearable devices. Such an approach would allow an -effective pre-screening process, reducing the number of clinical studies, reducing the number of products to be tested and the number of blood draws required in a clinical PK study Establishing such a prediction model might facilitate the longitudinal collection of data on product use and nicotine expression among consumers using nicotine products in their normal environments, thereby reducing the need for intrusive clinical studies while generating PK data related to product use in the real world.

An exploratory machine learning model was developed to predict changes in plasma nicotine levels following the use of an e-cigarette; from real time monitoring of physiological parameters and MLE to nicotine before, during, and after e-cigarette vaping. This preliminary study identified key parameters, such as heart rate (HR), heart rate variability (HRV), and physiological stress (PS) that may act as predictors for an individual’s plasma nicotine response (PK curve). Relative to baseline measurements (per participant), HR showed a significant increase for nicotine containing e-liquids and was consistent across sessions (intra-participant). Imputing missing values and training the model on all data resulted in 57% improvement from the original’learning’ data and achieved a median validation R^2^ of 0.70.

The study is in its exploratory phase, with limitations including a small and non-diverse sample size and reliance on data from a single e-cigarette product. These findings necessitate further research for validation and to enhance the model's generalisability and applicability in real-world settings. This study serves as a foundational step towards developing non-intrusive PK models for nicotine product use.

**Supplementary Information:**

The online version contains supplementary material available at 10.1186/s13040-024-00375-z.

## Introduction

Pharmacokinetic (PK) studies that measure nicotine levels in the blood plasma over time to estimate the amount of nicotine reaching systemic circulation, were first conducted in the 1980s [12, 27]. Since then, nicotine exposure from traditional tobacco products, such as cigarettes and smokeless tobacco/snuff, has been well characterised by the maximum nicotine concentration (Cmax), time taken to reach maximum concentration (Tmax) and overall nicotine dose (estimated from the area under the curve of plasma concentration against time, e.g., AUC60) [3]. These studies have shown that blood concentrations of nicotine rise quickly during smoking and peak upon completion of consumption [3].

Data provided by PK studies can yield fresh insights into how nicotine products are used, and the implications they have for abuse liability  [18], health effects [16], and addiction [27]. These studies, therefore, play an important role in the evaluation of new tobacco and nicotine products, including e-cigarettes, Tobacco Heating Products (THPs), and Nicotine Replacement Therapies (NRTs) [6, 10, 15].

However, PK studies are intrusive for the participants and must be conducted in a controlled clinical setting under medical supervision so are therefore both expensive and time-consuming. Additionally, blood sampling is generally conducted for a relatively short time post-puffing (e.g., 120 min). It is likely that, in a real-world environment, users would show different patterns of puffing and consumption behaviours, which may also change as they adapt to new products. In short, it would be useful to have a tool to predict long-term changes in nicotine PK under real-world conditions.

Methods of measuring nicotine exposure non-invasively have been developed, including mouth level exposure (MLE) using real-time optical obscuration [14, 21] and cigarette filter analysis [24, 28]. However, these approaches may be limited in their ability to predict the exposure of nicotine to, and absorption from, the respiratory tract because they do not consider non-inhaled puffs (mouth-spill) post-puff inhalation, exhalation patterns, or differences in the respiratory retention of individual aerosol constituents. Filter analysis is applicable only to cigarettes and provides an estimate of total rather than real-time exposure. Furthermore, optical obscuration requires participants to use the products through a puffing topography device in the clinic, which is used as a representative sample of real-world conditions. Lastly, MLE methods provide the maximum, total, or puff-by-puff quantity of nicotine that might reach systemic circulation, but do not consider the bioavailability of nicotine in the blood system. Therefore, novel non-intrusive methods of estimating nicotine exposure in a non-clinical setting are still required.

Through their ability to make high-quality predictions based on minimal training data, artificial Intelligence (AI) methods such as machine learning (ML) are being increasingly used to overcome time-consuming, intrusive, and costly data collection approaches in numerous fields, ranging from disease diagnosis to drug discovery [1, 7]. ML as a prediction approach has been used in clinical studies of smoking cessation, status, and addiction [9, 8, 26]. In particular, ML can train models for multiple endpoints. Furthermore, by using an iterative process to predict missing values that are put back into the model, ML algorithms can derive prediction models based on sparse datasets [30, 31]. As nicotine has several known physiological effects on heart rate (HR), blood pressure, and skin temperature [4, 17], we considered the possibility of identifying a set of physiological measures that could, via a prediction model, provide a “Digital Twin(s)” to mimic the output of clinical PK studies.

In this study, we have explored AI methods using physiological parameters such as surrogate measures of clinical PK data. We have developed a ML model that, based on physiological measurements, consumer puffing behaviour and MLE data collected via a connected e-cigarette device (ePen3), predicts nicotine uptake into the bloodstream. Our findings establish a platform for longitudinal monitoring of nicotine and tobacco product users in their everyday environment with minimal intrusion, while generating PK data related to product use in the real world. This new approach allows a ‘pre-screening’ of products based on key physiological parameters to help reduce the extent of intrusive PK testing required on study participants.

## Methods

The study was conducted in two phases: Phase 1 assessed the feasibility of the modelling approach by determining changes in physiological measurements during vapour product use and assessing initial nicotine levels predictions. Phase 2 incorporated observations and lessons learned from phase 1 to further improve prediction quality.

### Study participants

The study population comprised regular e-cigarette adult users who were also full-time employees of British American Tobacco (BAT). Participants had previously registered interest in the study, which was performed onsite at BAT, Southampton, UK. The inclusion criteria were general good health, minimum age of 21, and daily use of e-cigarettes for a minimum of 6 months. In addition, all participants were regular users of e-liquid with an average nicotine content of 12 mg/mL (Phase 1) and 18 mg/mL (Phase 2). Participants were excluded from the study if they were pregnant or breastfeeding, had an allergy or sensitivity to any of the ingredients in the e-liquids, or if they had an electronic pacemaker or other active implantable medical device.

For Phase 1 of the study, participants were asked to abstain from using any nicotine-containing products for 12 h prior to the study. For Phase 2 of the study, participants were asked to abstain from using any nicotine and/or caffeine-containing products for 12 h prior to the study. Participants were able to withdraw from the study at any time. After study completion, each participant received compensation for each session completed.

The study protocol and Informed Consent Form were approved by the Human Research Committee (HRC), British American Tobacco’s internal committee dedicated to ensuring all studies involving human subjects are carried out in accordance with the ethical principles outlined in the Declaration of Helsinki and other relevant guidelines.

### Study products puffing topography connected device

**Fig. 1:**
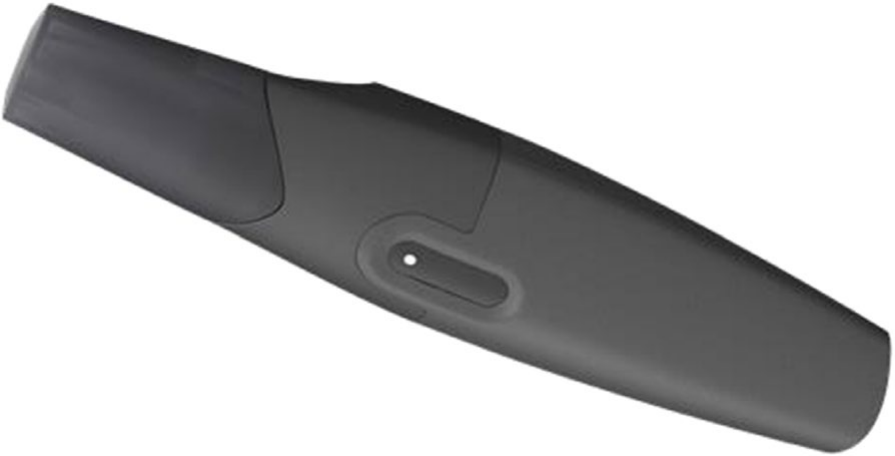
BAT’s Analytical Research Tool (ART)

An augmented e-cigarette device was used to measure puffing topography throughout the study. The Analytical Research Tool (ART) device (Fig. 1 and Supplementary Table S1) comprises an ePen3 vapour product [Nicoventures Trading Ltd, Globe House, London, UK] modified with the following sensors:

- flow sensor, to measure how the device is puffed and the temperature of the inlet air during puffing;
- temperature sensor, to measure the ambient air temperature;
- humidity sensor, to detect the external humidity.

Commercial ePen3 e-liquids (Dark Cherry, 0 mg/mL or 12 mg nicotine cartridges) were used with the ART device [BAT, London, UK] (Supplementary Table S2).

This study primarily examines the Dark Cherry flavour, the most popular-eliquid in the UK, to demonstrate a concept that adheres to UK market regulations. In contrast to the Frosina et al. study conducted in Canada, where nicotine levels are allowed up to 50 mg/mL, this study aligns with the UK’s legal limit of 20 mg/mL. To ensure legal compliance, the product was appropriately adapted for a UK study. The purpose of this manuscript is a proof of concept. A subsequent study, which is outside of the scope of this manuscript, will rigorously test hypotheses and validate the findings presented here. This follow-on study will involve a single cohort of participants who will undergo blood draws and physiological monitoring to validate the methodology and results from this initial study.

### Physiological measurement devices

**Fig. 2:**
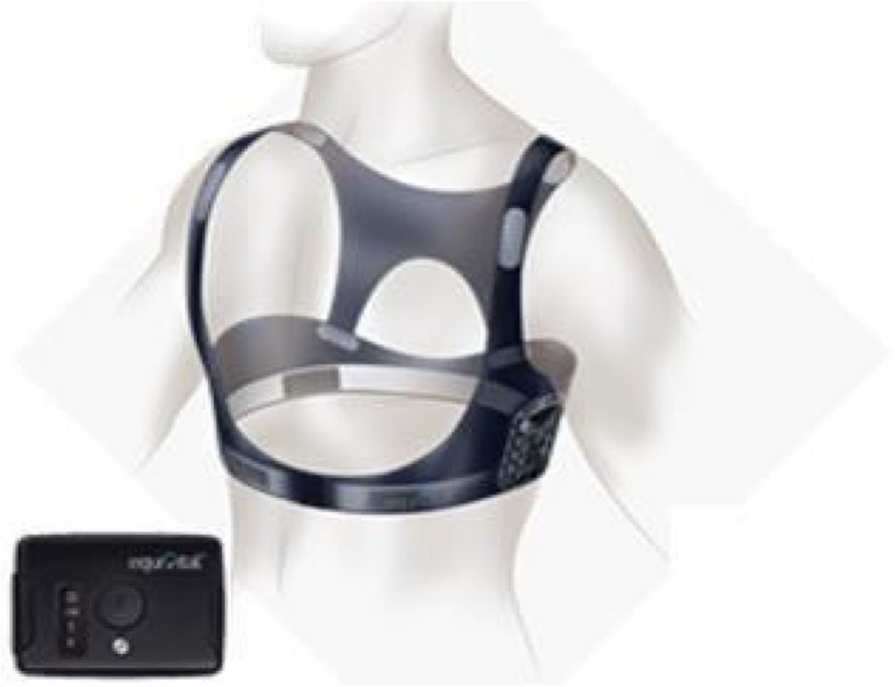
Equivital’s Eq. 02 + LifeMonitor

For all participants (with the exception of one), physiological data were collected using an Eq. 02 + LifeMonitor [Equivital, Cambridge, UK] (Fig. 2). This is a chest-based, Food and Drug Administration (FDA) registered, physiological monitoring system, which has been extensively used in research and clinical studies and has been independently validated [22]. In one case, where a suitable belt size was not available, data were collected using the FDA cleared Bittium Faros 180 Electrocardiogram (ECG) Monitor [Bittium Corporation, Oulu, Finland].

The Eq. 02 + LifeMonitor system recorded ECG and respiration waveforms (at 256 Hz and 25.6 Hz, respectively) and provided outputs for heart rate (HR), breathing rate (BR), and skin temperature (based on a clinical grade infrared thermometer reporting skin temperature every 5 s). In addition, 50% of participants wore an auxiliary oxygen saturation sensor (Nonin XPOD 3012LP pulse oximeter) which also recorded photoplethysmography (PPG) waveforms at 100 Hz [Nonin, Plymouth, Minnesota, USA]. The other 50% wore an auxiliary Galvanic Skin Response (GSR) sensor which measured skin conductance in micro-Siemens at 16 Hz [Equivital, Cambridge, UK]. The Bittium Faros 180 monitor collected ECG data only with a sampling frequency of 500 Hz (See Supplementary Fig. 7).

ECG data from the Eq. 02 + LifeMonitor system and the Bittium Faros 180 monitor were then processed through B-Secur’s FDA 510(k) cleared HeartKey® software library [B-Secur, Belfast, UK] to extract the following health and wellness metrics: HR, HRV, and Physiological Stress (PS).

### Study protocol

Each study session lasted approximately 90 min. Prior to data collection, participants were fitted with the physiological monitoring belt (Eq. 02 + LifeMonitor or Bittium Faros 180 Monitor) and the use of the modified vapour product (ART) was explained. The ART e-liquid was weighed immediately before and after use to determine the Device Mass Loss (DML), which enabled nicotine exposure to be estimated.

Data collection was divided into three stages: pre-vaping, vaping, and post-vaping. Throughout the monitoring period, participants were seated in a calm environment to minimise changes in physiological parameters resulting from physical movement and other external factors. Baseline physiological data were recorded during the pre-vaping stage. In the vaping stage, participants were instructed to use the ART study product with designated e-liquid for 10 min, taking 1 puff every 30 s, resulting in a total of 21 puffs. In the post-vaping stage, physiological data were continuously recorded for up to 60 min.

Although physiological measurements were constantly measured throughout the study, specific timepoints before, during, and after vaping that aligned with the measurement points in the historic PK dataset were used for data analysis and to facilitate direct comparisons (Supplementary Table S3). Based on observations from Phase 1, the baseline data collection period was increased to 50 min before vaping in Phase 2 to better account for natural fluctuations in HR and to establish a more accurate baseline. In addition to physiological measurements, participants completed a short questionnaire in Phase 1 that included questions about caffeine use and exercise. In Phase 2, additional questions regarding daily nicotine intake, smoking habits, and length of nicotine product use, were included in the questionnaire.

### Study measures

**Fig. 3:**
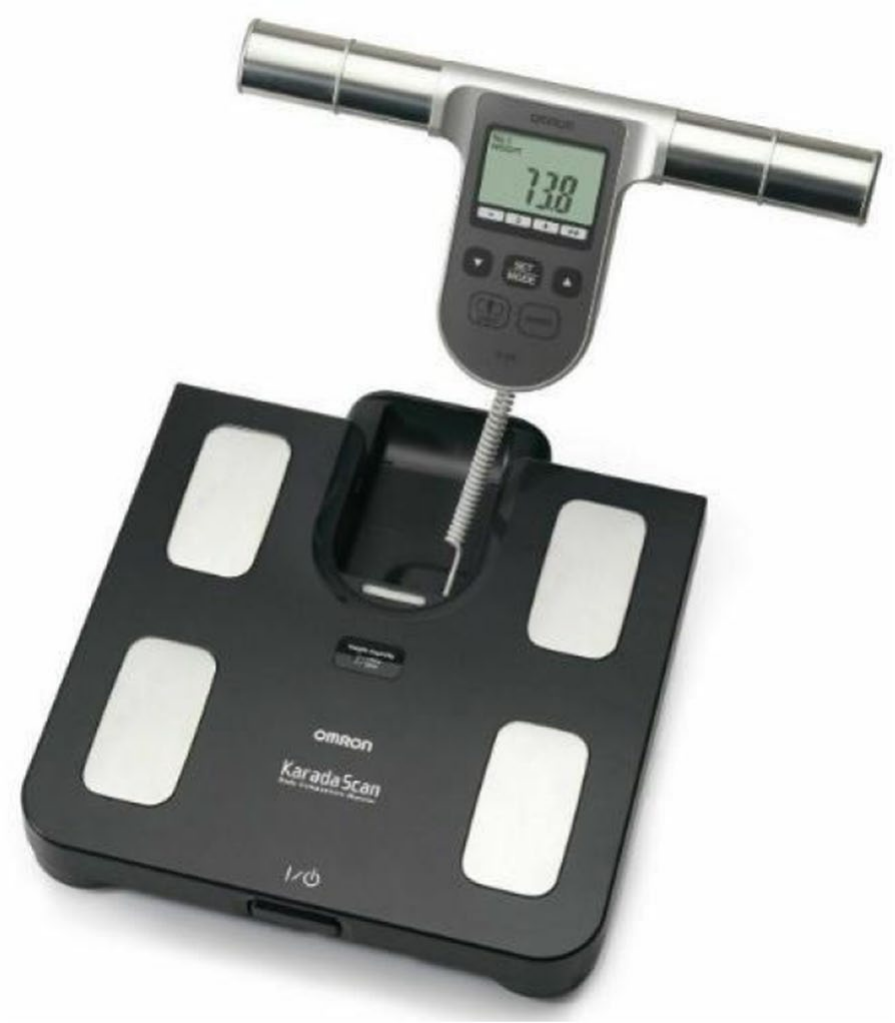
Omron’s Body Composition and Body Fat Monitor (Model BF511)

Age, sex, height, and weight were recorded for each participant. In Phase 1, body mass index (BMI), body fat percentage, and muscle percentage were measured using Smart Scales. In Phase 2, visceral fat (VF) percentage and resting metabolism (RM) were additionally measured by using an Omron Body Composition and Fat Monitor (Model BF511) (Fig. 3).

Data collected on the Eq. 02 + LifeMonitor system included ECG and respiration waveform data, skin temperature, GSR, oxygen saturation, and associated HR, and BR data. The raw ECG data were processed through the HeartKey® software library to extract values for HR, HRV, and PS for input into the prediction model. Data collected from the ART device included puff duration, puff volume, and DML. Qualitative data were collected from questionnaires completed before and after the monitoring period.

### Historic nicotine PK data for modelling

A historic, nicotine PK data set was used in the study as a ‘learning’ dataset to initially train the ML model for predicting plasma nicotine levels [13]. The ‘Clinical data were obtained during a standard blood draw trial from 30 adult smokers, each using four products (a conventional cigarette, and an e-cigarette with three different e-liquids) with blood samples taken for 90 min from the start of product use. For the historical PK dataset, a period of 8 h of abstinence from nicotine-containing products before the first daily blood draw was observed.

In addition to the PK values (C_max_, T_max_, AUC_60_) for the 120 participant/product combinations, this data set contained participant characteristics (biological sex, age, and BMI), product information (device type and nicotine strength), puffing type (ad libitum or fixed puff), and blood draw timings, among other descriptors. To better understand any potential factors that could affect the accuracy of the models' predictions, the blood draw time was converted into month, day, and hour. This was done to determine whether any variability in the blood draw measurements could be explained by systematic or behavioural changes, such as those related to the study parameters. The extracted PK data included the maximum concentration of plasma nicotine (C_max_), time taken to reach C_max_ (T_max_), area under the concentration–time curve from the time of dosing to the time of the last sample collection (AUC_60_), along with plasma nicotine concentrations at specific time points relative to nicotine product use (0 (baseline), 5, 8, 10, 15, 30, and 60 min). The study timepoints are grouped into three distinct stages, 1) pre-vaping (50 min baseline), 2) vaping (5, 8, 10 min), and 3) post-vaping (15, 30, 60 min).

In this study, time was not used as an input variable. To enable analysis of the physiological and nicotine data measured at seven distinct time points throughout the study, a data format appropriate to the problem at hand was adopted. Rather than representing each study participant as having seven separate data points (rows) in the dataset, a single data point for each participant was created. This was achieved by expanding each feature being measured into seven separate columns in the dataset. For example, the heart rate measurement was decomposed into seven columns, representing heart rate measured at different time points such as heart_rate_0 mins, heart_rate_5 mins, heart_rate_8 mins, and so on. This formatting approach enabled simplification of the dataset while retaining all the necessary information for analysis and allowed us to use a wider range of statistical methods for modelling and inference.

### Data collation and analysis

All data, including physiological data, device usage data, participant questionnaire data, and historic nicotine PK data (existing clinical were entered into the data collation and analysis program Zizo [Zizo Software, Milton Keynes, UK]. This platform comprises an in-memory pattern database for the storage of large datasets (Zizo DB), a processing tool to integrate, clean and transform the data (Zizo Pathway), and a visualisation and exploration tool (Insight). The platform integrated the various datasets and generated a spreadsheet for each participant containing numerous descriptors, including participant characteristics (sex, age, height), experimental parameters (puffing type, nicotine level, product), and physiological markers (HR, BR, oxygen saturation, HRV) recorded at multiple time points across the study.

Data were analysed by B-Secur and Equivital using a range of analysis tools including Matlab and Excel respectively, to identify trends and potential correlations between vaping and physiological response.

### Modelling methodology

**Fig. 4:**
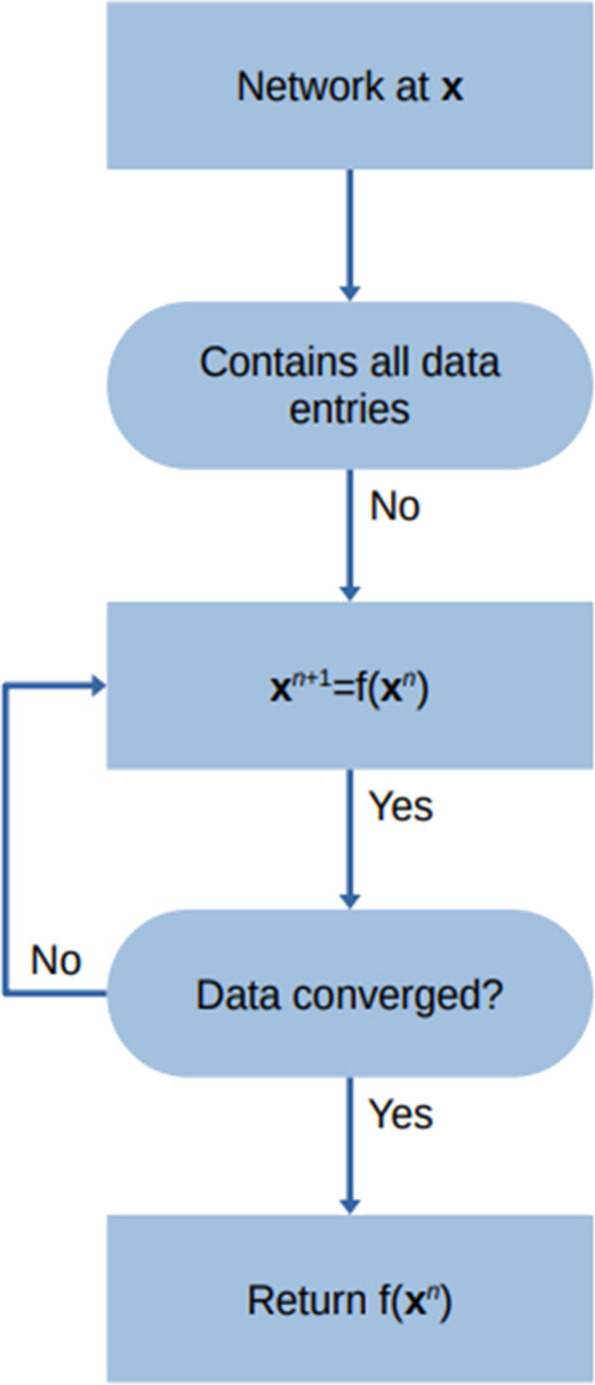
A graphical illustration of the machine learning method for sparse data imputation

The model generated to predict plasma nicotine levels from physiological parameters was developed with Alchemite™ [Intellegens, Cambridge, UK], an ML algorithm used to handle sparse input data in a variety of fields [19, 23, 30, 31]. Alchemite™ distinguishes itself by its robust methods to prevent overfitting, a common pitfall in machine learning, especially in datasets with sparsity and significant noise. It achieves this through advanced techniques such as automated feature selection, Bayesian optimisation methods, and assembling strategies, which collectively enhance the model's generalisability and predictive accuracy. A graphical representation of the sparse imputation fitting procedure of the Alchemite™ machine learning algorithm is illustrated within Fig. 4.

The initial model was trained using the existing clinical dataset, including C_max_, T_max_, AUC_60_, and plasma nicotine concentrations at specific time points relative to nicotine product use (0 (baseline), 5, 8, 10, 15, 30, and 60 min) [13]. C_max_ and plasma nicotine concentration values were log-transformed to reduce the relative effect of high values on the variance, improving the model’s ability to generalise to unseen data.

To maximise the amount of data used during training and to build a more generalised model, a fivefold cross-validation approach was employed, in which the training dataset was partitioned into five randomly selected subsets. A ML model was subsequently trained on each subset, except for one, which was used as the validation set, and performance was measured by making predictions and calculating the coefficient of determination (*R*^2^):

$$R_2=1-\frac{\sum_i(ypredi-yobsi)^2}{\sum_i(y^{{obs}_i-}\overline{{\overline y}}\;ob\overline{\overline{\overline{\overline s)}}}}2,$$

where *y*_*i*_^*obs*^ is the *i*^*t*ℎ^ observed value and *y*_*i*_^*pred*^ is the corresponding prediction. The closer the value of *R*^2^ to 1, the higher the predictive ability of the model. To identify the best performing set of model hyperparameters, sequential model-based optimisation was performed using the Tree-structured Parzen Estimator (TPE) [5]. Sixty models were sequentially trained, with the aim to maximise the median *R*^2^ across all targets in the validation sets. Note that all *R*^2^ measures reported are from validation sets: we do not record the training-set fit performances, as validation performance is a more reliable estimate of model performance on future, unseen, test data.

Note, only columns that were at an equivalent or earlier time point to the plasma nicotine concentration being modelled were permitted as inputs. This ensured that only inputs that were available at test time would be used in making a prediction, mimicking the real application of the model in a potential clinical setting.

The present study was conducted to explore whether various physiological parameters, such as HR and BR, might be used as surrogate measures for nicotine plasma levels during and after use of a vapour product. Ultimately, establishing such correlations might facilitate the collection of data on product use and nicotine expression among consumers using nicotine products in their normal environments, thereby reducing the need for intrusive clinical studies.

### Identifying physiological trends during vapour product use (Phase 1)

Phase 1 of the study explored whether there was a direct correlation between various physiological metrics and nicotine exposure and tried to pinpoint measures that might best contribute to a model for predicting plasma nicotine levels following the use of a vapour product. To explore additional inputs into the prediction model, user puffing behaviour, including puff duration, puff volume, and DML were also collected. In order to ensure consistency with the historical PK data set, the period of abstinence from nicotine-containing products before the first daily blood draw was set at 8 h.

### Identifying additional physiological inputs and improving model performance (Phase 2)

Phase 2 further explored additional physiological inputs, such as HRV, PS, and BR, and their impact on model performance. More participants were utilised to capture greater variance within the data to improve model performance and generalisability, thereby building a stronger model with a more robust study design utilising a control sample. Based on the historical PK and Phase 1 model findings, the period of abstinence from nicotine-containing products prior to the first daily blood draw was extended to 12 h. The details of this change are discussed in the 'Results and Discussion' section that follows.

## Results and discussion

The present exploratory study was conducted to explore whether various physiological parameters, such as HR and BR, might be used as surrogate measures for nicotine plasma levels during and after use of a vapour product with the aim of identifying a set of physiological measures that might, via a prediction model, provide a “digital twin” to mimic the output of clinical PK studies. Ultimately, establishing such a prediction model might facilitate the longitudinal collection of data on product use and nicotine expression among consumers using nicotine products in their normal environments, thereby reducing the need for intrusive clinical studies while generating PK data related to product use in the real world.

### Feasibility study (Phase 1)

Phase 1 of the study tested the feasibility of our approach. Physiological measures and product use parameters were evaluated among individuals using a vapour product and used to inform an initial prediction model trained on a historic dataset of clinical PK data [13].

#### Physiological trends during vapour product use

In total, 13 adult e-cigarette users were recruited to use the ART device with 12 mg/mL nicotine e-liquid (Table 1). The ages of the participants ranged from 27 to 43 years, with 62% (8/13) of the study population being male. Twelve participants wore the Eq. 02 + LifeMonitor system, with additional sensors for GSR (*n* = 6) and oxygen saturation (*n* = 6); one participant wore the Bittium Faros 180 monitor, which collected only ECG data.

**Table 1:** Characteristics of participants in Phase 1 using ART with 12 mg/mL nicotine e-liquid

**ID**	**Age (years)**	**Weight (kg)**	**Height (cm)**	**BMI** (kg/m^2)	**Fat (%)**	**Muscle (%)**	**Sex**
30	32	71.4	167.0	25.6	26.7	35.7	M
28	30	99.4	180.0	30.7	32.0	32.5	M
32	28	77.0	160.0	37.1	41.0	21.0	F
31	27	90.7	180.5	27.8	30.0	33.8	M
29	30	74.6	170.5	25.7	24.6	37.2	M
27	39	112.3	172.5	37.7	41.4	26.8	M
26	28	56.3	168.0	19.9	29.0	28.2	F
24	34	85.4	162.0	32.5	48.3	22.5	F
17	27	76.8	176.0	24.8	24.4	37.1	M
33	32	73.0	176.0	23.6	35.3	27.6	F
24	43	57.0	172.0	19.3	28.1	27.8	F
35	28	79.1	179.0	25.8	27.2	34.7	M
36	35	81.4	175.0	25.4	20.3	39.4	M

Over the 90 min study session, user puffing behaviour data were collected for all 13 participants. Notably, DML, which gives an estimate of MLE to nicotine, varied widely across the participants from 0.04 to 0.33 mg/mL (Supplementary Table S4).

Physiological data were also successfully collected for all 13 participants, enabling the exploration of correlations among the various physiological measures. Consistent with previous studies [11], HR positively correlated with BMI. However further investigation is required to establish the extent to which this correlation is accurate. Fairly large intra-participant variations in HR were noted over the 10 min baseline collection period, suggesting the need to establish the resting HR over a longer baseline period for each participant.

**Fig. 5:**
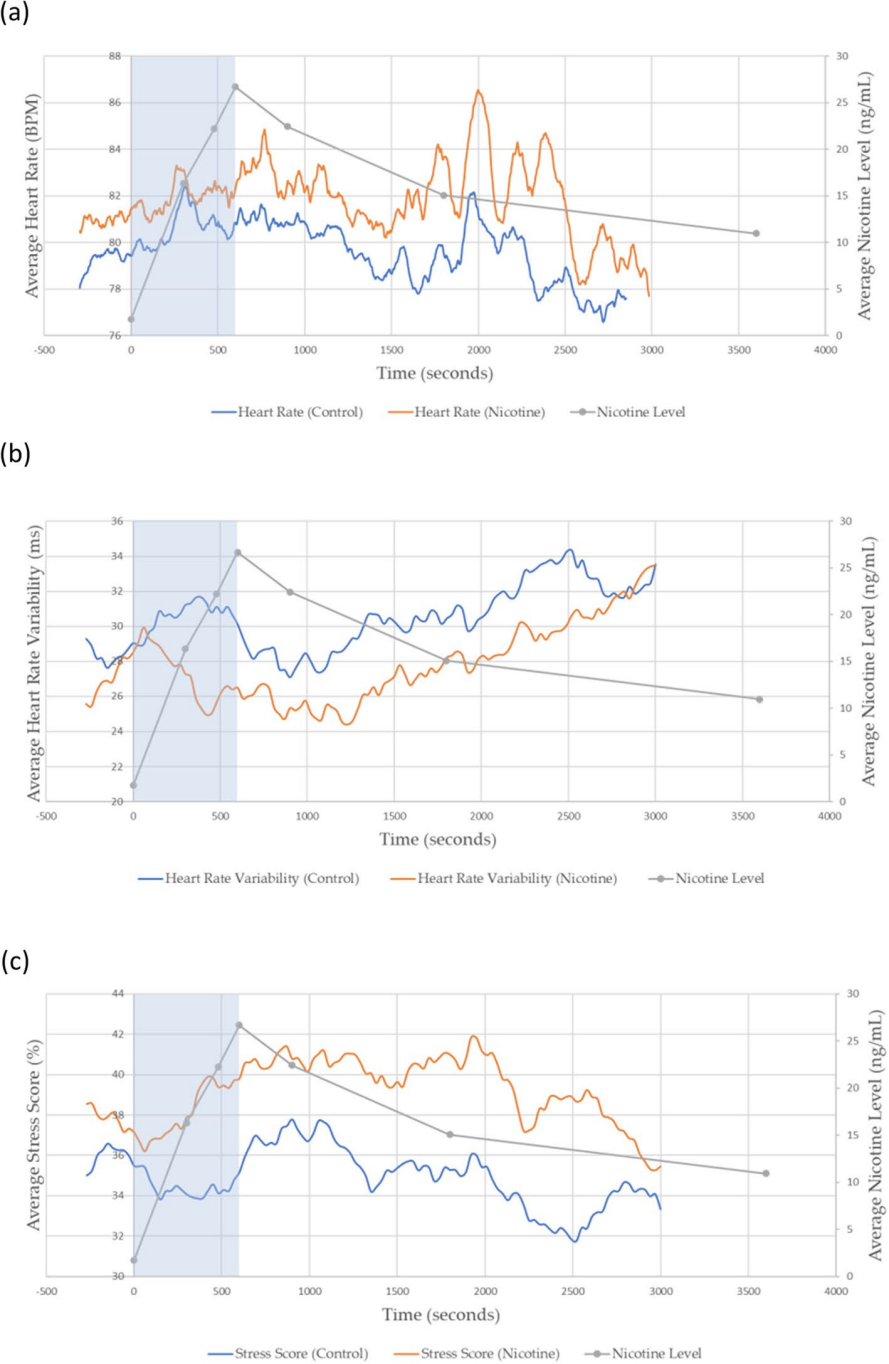
Comparison between average plasma nicotine levels derived from historic nicotine PK data **a** Average Heart Rate, **b** Average Heart Rate Variability, and **c** Average Physiological Stress between nicotine and control (0 mg/mL nicotine) vapour product use. A 5-min baseline was employed before the beginning of the session. The ‘during-puffing’ stage is indicated within the blue box

As expected from previous studies [2, 29], there was an increase in mean HR during the vaping session, which was proportional to the trend in predicted nicotine levels and the plasma nicotine levels derived from the historic PK data (Fig. 5 (a); *p*-value for correlation being significant: 6 × 10^–50^). A similar correlation was observed for mean PS (Fig. 5 (c); *p*-value for correlation being significant: 4 × 10^–9^), while HRV showed an inverse correlation with estimated nicotine levels (Fig. 5 (b); *p*-value for correlation being significant: 8 × 10^–7^). However, BR, skin temperature, and GSR displayed little or no change, during the vaping session. Changes were noted in oxygen saturation, but they were too coarse and did not seem to correlate with nicotine exposure. Thus, among the physiological measures recorded, HR, HRV, and PS showed the most promise as potential predictors of nicotine levels during vapour product use.

To obtain preliminary control HR data, the study was repeated on four participants (Participant ID 17, 27, 29, 32) who used ART with the same flavour e-liquid (Dark Cherry) but without nicotine (0 mg/mL). Control group participants were blinded to nicotine content. Three participants wore the Eq. 02 + LifeMonitor system (GSR, *n* = 1; oxygen saturation, *n* = 2) and one participant wore the Bittium Faros 180 monitor (ECG data only). The biometric data showed that the PS across control participants was not significantly correlated with predicted nicotine levels during the vaping session at the 5% level (p-value for significant correlation: 0.08), but no evidence was obtained that HR and HRV were uncorrelated with predicted nicotine levels using the control e-liquid (Fig. 5; p-values for significant correlation 2 × 10^–49^ and 4 × 10^–4^ respectively). However, a clear systematic shift between the control and nicotine groups is observed for the three physiological responses plotted in Fig. 5a-c. This highlighted the importance of collecting extended baseline measurements to be able to correct for the uncertainty in measurements due to limited vaping time and a longer abstinence window before data collection to avoid nicotine remaining in the blood at the beginning of the vaping session.

### Initial nicotine plasma prediction model

Numerous AI modelling algorithms have been reported in recent years. In selecting a modelling approach to predict plasma nicotine levels, the main consideration was the high proportion of missing values (≈82%) which resulted from merging the historic clinical PK dataset with Phase 1 and 2 data. An imputation program based on ML was therefore considered to provide a good starting basis. Specifically, an imputation model uses all existing data (descriptors and target endpoints) as an input and fills in the missing values by predicting them from the data that is present [19]. Furthermore, such model enables prediction uncertainty to be calculated, which means that the quality of the prediction can be improved by focusing on the most confident results. The imputation algorithm Alchemite™ uses an iterative process to predict missing values and subsequently derive prediction models based on sparse data sets containing as few as 6.3% of data points [30, 31]. Although developed for materials science applications [30], it has been shown to outperform other computational approaches in the fields of structure–activity relationships [31], drug discovery [19, 20], rat PK [25], and sensory analyses [23]. Alchemite™ was selected based on this flexibility, coupled with its ability to be retrained as more data becomes available [19].

A preliminary model was built using the historic clinical PK dataset. Descriptors included participant characteristics (biological sex, age, and BMI), blood draw timings (day, month, and hour), product information (device type and nicotine strength), and puffing type (ad libitum or fixed puff). The targets were *C*_max_, *T*_max_ and nicotine plasma levels recorded at seven timepoints (0, 5, 8, 10, 15, 30, and 60 min) for each participant. The preliminary model achieved reasonable accuracy overall, with a cross-validation performance of *R*^2^ = 0.446, in line with results from modelling studies in related fields, such as prediction of rat PK parameters [25], and performed better at post-vaping time points (15, 30, and 60 min) than at vaping time points (5 and 8 min) (Supplementary Figure S1).

Subsequently, the plasma nicotine prediction model was retrained with the additional Phase 1 data, comprising 0 mg/mL nicotine eliquid (control), as well as new descriptors, including participant characteristics (muscle percentage, body weight percentage), experimental parameters (puffing volume, product nicotine level, DML), and physiological markers (HR, HRV, BR, oxygen saturation) taken at seven time points (0, 5, 8, 10, 15, 30, and 60 min) for the nine targets (T_max_, C_max_, and predicted nicotine concentration at (0, 5, 8, 10, 15, 30, and 60 min). Incorporating the Phase 1 data in the model resulted in an 18% improvement in predictive accuracy on validation data (*R*^2^ = 0.526) over the preliminary model using only the existing clinical data set (Supplementary Figure S6 and Supplementary Figure S8). The increase in data has helped the ML algorithm to understand underlying trends better by ‘seeing through the noise,’ while the new physiological measurements reduced the amount of unexplained variance. In addition, key descriptors that contributed most strongly to the model’s predictions were identified using feature importance values, such as a change in HR from baseline ($\Delta$ HR) and the participant’s age (Supplementary Figure S2). Furthermore, areas within the domain that are known to be important but were not covered in the original clinical dataset, such as PS, HRV, and puffing volume were highlighted. Moreover, the feature importance analysis of the model yielded intriguing results as the variables of month, hour, and day were identified as significant predictors of blood nicotine levels. In light of this, the Phase 2 study implemented measures to reduce the presence of nicotine in blood plasma. Specifically, the abstaining time from nicotine-containing products was increased from 8 to 12 h, and the consumption of caffeine-containing products within this timeframe was curtailed. In addition, more stringent study protocols were implemented. These steps were taken based on observations made during the historical and Phase 1 studies. Notably, the study uncovered significant findings regarding the month variable, potentially indicating behavioural changes during the holiday season as data was collected in December 2020 and January 2021. Additionally, the day of the blood draw was found to be significant to a lesser extent, which could be attributed to behavioural changes over the course of the week. To ensure that the prediction model could be applied to new data, the study hour, day, and month were excluded from future analyses. This simplification not only enhances the model's interpretability and generalisability but also ensures that time is not used as an input. As a result, the Phase 2 model relied solely on demographic and physiological data as inputs.

**Fig. 6:**
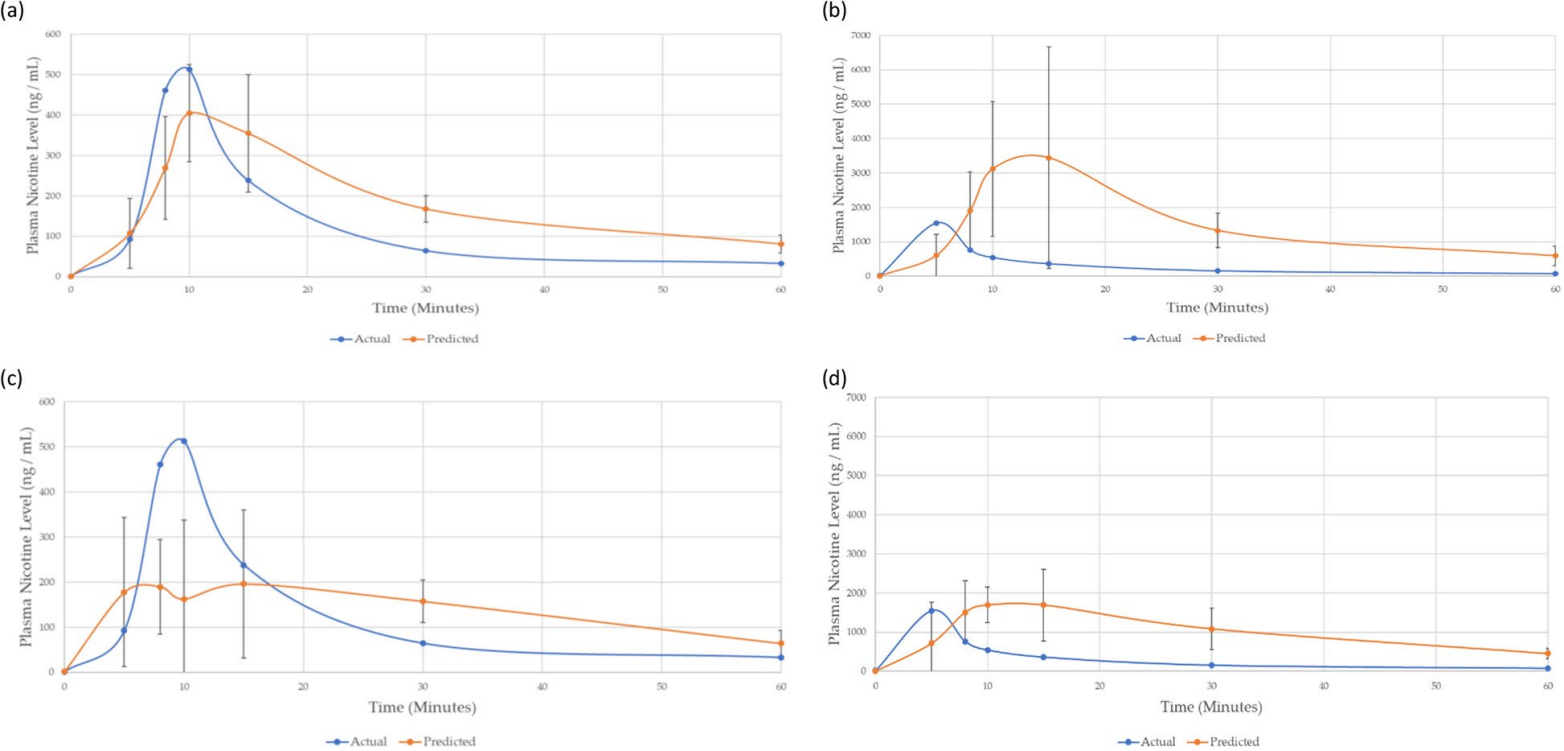
Predicted and actual plasma nicotine levels for an individual using an e-cigarette under fixed and ad libitum puffing regimes; the predicted participants data were not contained within the models’ training set. The predictions from the existing clinical model for fixed puff are shown in (**a**) and ad libitum in (**b**). The predictions for the revised Phase 1 model for fixed puff are shown in (**c**) and ad libitum in (**d**)

To evaluate performance, both the initial model built on historic nicotine PK data and the revised model incorporating Phase 1 data were used to predict plasma nicotine levels for a participant from the existing clinical study who was not included in the training set (Fig. 6). All predicted plasma nicotine values were accompanied with a corresponding uncertainty value, which indicates the model’s confidence in its prediction. The predicted levels showed reasonable agreement with the actual nicotine concentrations measured in the participant’s plasma (Fig. 6). Predictions with larger error between the actual plasma nicotine value also had higher uncertainty. Therefore, the model was less confident in those predictions. It is pertinent to note, that uncertainty can be reduced, and prediction accuracy improved by acquiring more participant data, as well as identifying better predictors for modelling.

Overall, the findings from Phase 1 supported the feasibility of using a ML imputation algorithm and physiological measurements including HR and PS to predict plasma nicotine levels; in particular, HR was identified as a strong predictor of plasma nicotine levels. Moreover, a key observation (Fig. 6) was the wide variability in individual baseline HR, indicating that more robust baseline physiological data would be needed to obtain a better understanding of “normal” resting HR and to improve estimates of HR during use of the vapour product. In relation to this, the intra-participant reproducibility of HR measurements should be established and data from a larger set of participants using non-nicotine (control) e-liquids should be collected.

### Revised plasma nicotine prediction model (Phase 2)

To improve any prediction model, the quantity, quality, and diversity of data are critical to allow the model to learn from the widest possible spectrum of cases. Therefore, in Phase 2 of the study, more data, including new physiological measures and data from e-liquids with different strengths of nicotine, were obtained for a larger number of participants. Based on the above findings on average HR (Fig. 5a), the baseline period for data collection was increased from 10 to 50 min. Participants were also asked to abstain from both nicotine and caffeine for 12 h before the first blood draw to reduce any trace amount of blood nicotine. These new datapoints were incorporated into the initial model to produce a revised ML model for predicting plasma levels during vapour product use.

Among the 19 participants recruited in Phase 2, 16 used ART with 18 mg/mL nicotine, 1 with 12 mg/mL nicotine, 1 with 6 mg/mL nicotine, and 1 with 0 mg/mL nicotine (based on personal preference / ‘normal use’). To expand the control data, the 18 participants using nicotine-containing e-liquids also attended a second puffing session, in which they used ART with 0 mg/mL nicotine (participants were blinded to this). In addition, one participant (ID 39) took part in three vaping sessions using ART with 18 mg/mL nicotine e-liquid to establish the reproducibility of the physiological measurements. Lastly, additional physical data, including RM and percentage VF were collected via smart scales (Table 2). More extensive data on exercise level, caffeine and nicotine intake, and the length of nicotine product use, among others, were also collected via the participant questionnaire; these data were not used in the modelling but were gathered to help build greater context around the observed trends.

**Table 2:** Characteristics of participants in Phase 2 using ART with 0–18 mg/mL nicotine e-liquid

ID	Age (years)	Weight (kg)	Height (cm)	BMI	Fat (%)	Muscle (%)	Sex	Visceral Fat (%)	RM (kcal)
17	28	78.7	176	25.3	25.6	26.2	M	8	1747
18	30	105.1	172.5	35.3	45.5	19.9	M	19	1891
22	46	89.4	166	32.4	50.6	20.8	F	10	1595
23	40	58.6	168	20.8	29.0	29.2	F	4	1317
24	34	85.4	162	32.5	48.3	22.5	F	8	1536
26	29	56.7	168	20.1	29.4	28.0	F	8	1297
27	40	111.7	175.5	36.3	39.2	27.8	M	19	2145
28	30	96.5	180	29.8	29.4	33.9	M	11	1981
29	30	73.6	170.5	25.3	22.9	38.2	M	8	1692
31	27	92.7	180.5	28.5	30.3	33.6	M	11	1932
32	27	76.4	160	29.8	44.3	24.3	F	7	1441
33	32	76.7	176	24.8	35.2	28.3	F	5	1542
34	43	58.5	172	19.8	27.4	29.3	F	3	1339
35	28	79.8	179	24.9	20.9	39.0	M	7	1772
36	33	79.8	175	26.1	27.0	35.0	M	9	1749
37	23	63.6	177	20.3	24.4	32.4	F	3	1425
38	26	82.4	173	27.5	25.0	37.4	M	10	1821
39	37	68	171	23.3	21.7	37.5	M	7	1581
40	29	71.6	180	22.1	15.9	41.4	M	4	1655

**Fig. 7:**
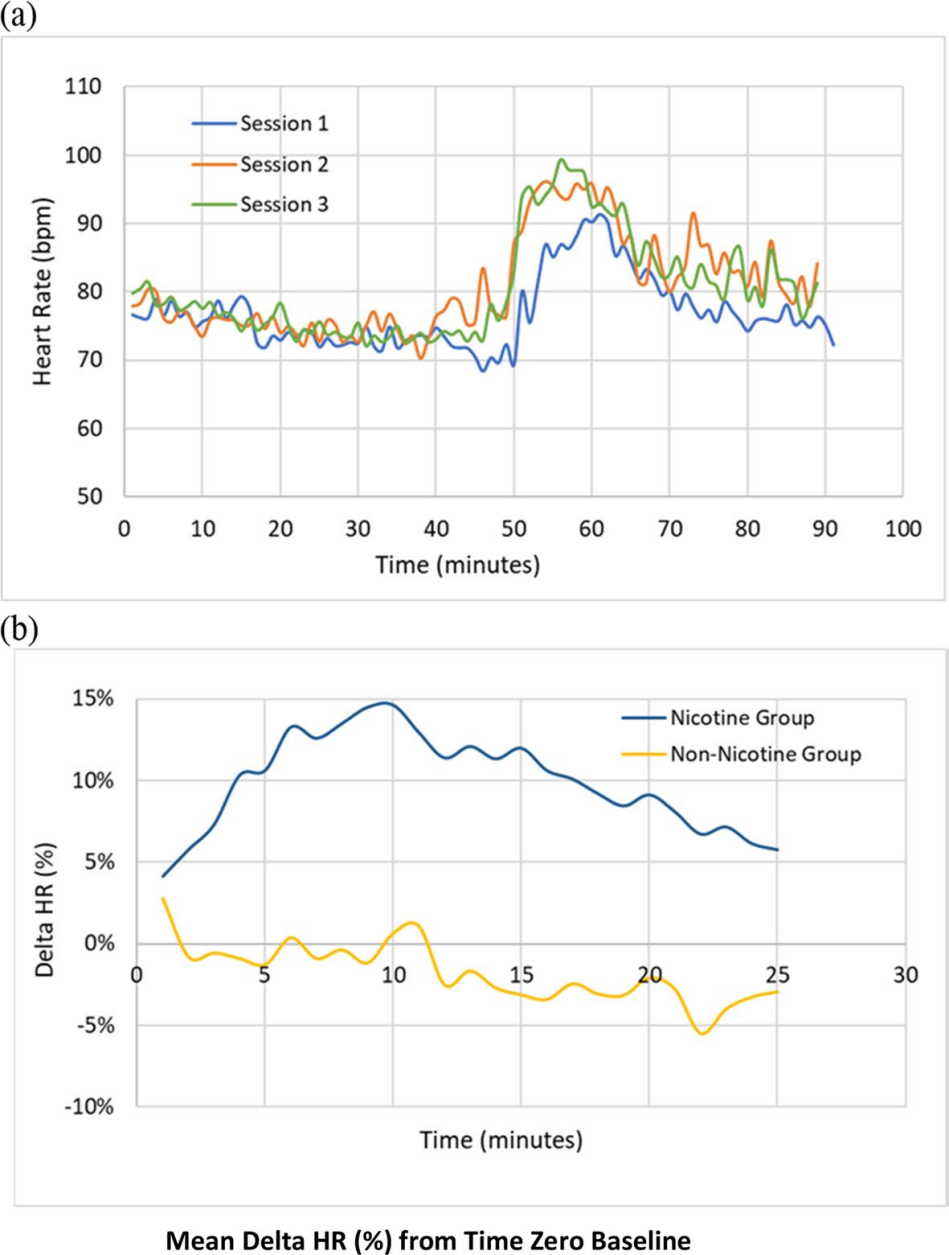
**a** Reproducibility of HR measurements in one participant. **b** Mean percentage change in HR from baseline in nicotine and non-nicotine groups

Biometric data were successfully collected for the 19 participants, all of whom wore the Eq. 02 + LifeMonitor system (GSR, n = 10; oxygen saturation, n = 9). The reproducibility of the physiological measurements was initially tested by conducting three nicotine vaping sessions for one participant. Figure 7a shows that HR for this individual followed a similar trend in all three sessions. The subsequent collection of physiological measurements for 50 min before the start of the vaping stage enabled a more confident determination of the mean change in HR from the baseline. ΔHR (all participant average results), peaked at the end of the vaping session (10 min) in the nicotine group but remained relatively close to 0 throughout the 10 min puffing session in the non-nicotine group (Fig. 7b and Supplementary Figure S3).

Regarding other physiological measurements, BR remained unchanged in both the nicotine and control groups (Supplementary Figure S4). Contrary to Phase 1, where little change in GSR was observed, GSR peaked at approximately 20 min after the start of the vaping session for seven participants (Supplementary Figure S5). A possible explanation for this difference was the closer adherence to abstinence from both nicotine and caffeine for 12 h prior to data collection in Phase 2, as determined from the participant questionnaires (SQ1). PS and HRV showed the same trends as observed in Phase 1 of the study – namely, PS increased and HRV decreased with the trend in plasma nicotine levels (data not shown).

Like Phase 1, DML, indicating MLE to nicotine, ranged widely from 0.05 to 0.23 mg/mL for the 18 mg/mL nicotine e-liquid (n = 16), and was 0.09 mg/mL and 0.11 mg/mL for the 12 and 6 mg/mL nicotine e-liquids, respectively. DML for the nicotine-free e-liquid also ranged widely from 0.07 to 0.26 mg. Therefore, no direct link could be determined between DML and change in physiological measurements which may be due to participants taking a puff but not inhaling, which is a common consumer behaviour when using inhalation nicotine products (Supplementary Table S4).

The existing clinical data, Phase 1, and Phase 2 data were compiled into a final global dataset with 153 descriptors, 9 targets, and significant sparsity (~ 82% missing). The descriptors consisted of 7 participant characteristics (age, BMI, percentage fat/muscle/visceral fat), 6 experimental parameters (puffing type, nicotine level, type of product), and 20 physiological markers taken at seven time points (HR, oxygen saturation, HRV). The targets were *C*_max_, *T*_max_, and seven plasma nicotine concentrations at baseline (0), 5, 8, 10, 15, 30 and 60 min from the start of vaping.

**Fig. 8:**
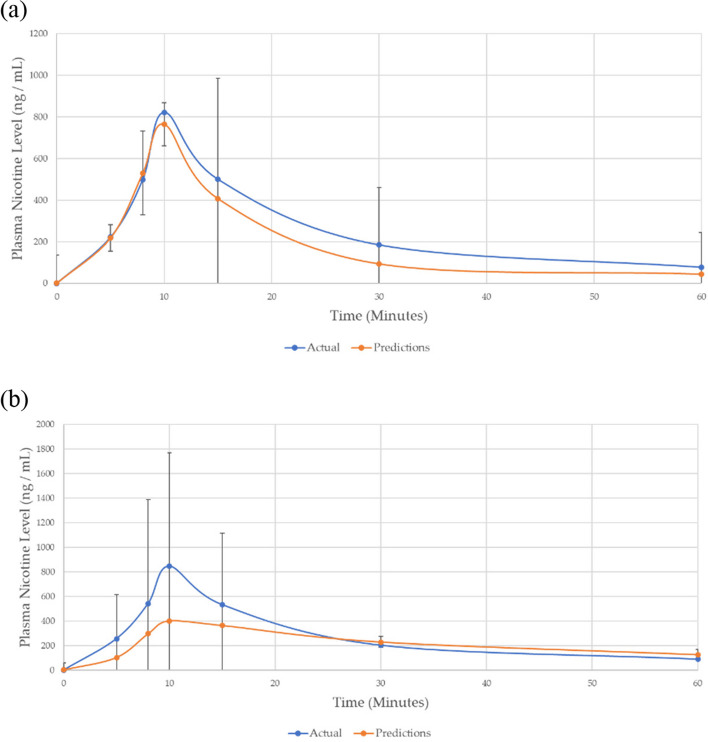
**a** Fixed puff and **b** Ad Libitum plasma nicotine concentration PK-curve predictions for a participant whose data were removed from the training set

To give an indication of the modelling quality using this final dataset, performance on unseen data for one participant was removed from the global dataset and their plasma nicotine levels were predicted from their physiological data alone. Figure 8 shows that there was close correspondence between the participant’s predicted and their actual plasma nicotine levels determined in the PK study for fixed puffing, although the prediction of C_max_ was somewhat higher for ad libitum. It is believed that puffing was harder to predict for ad libitum due to the greater variability in consumer puffing behaviour. Note, the original focus of this study was only on fixed puff and not ad libitum.

In a follow up study, the model performance on unseen data could be further improved by either applying stricter environmental controls to minimise the influence of other effects, such as, movement, distractions etc. or by including this information as inputs for modelling.

**Fig. 9:**
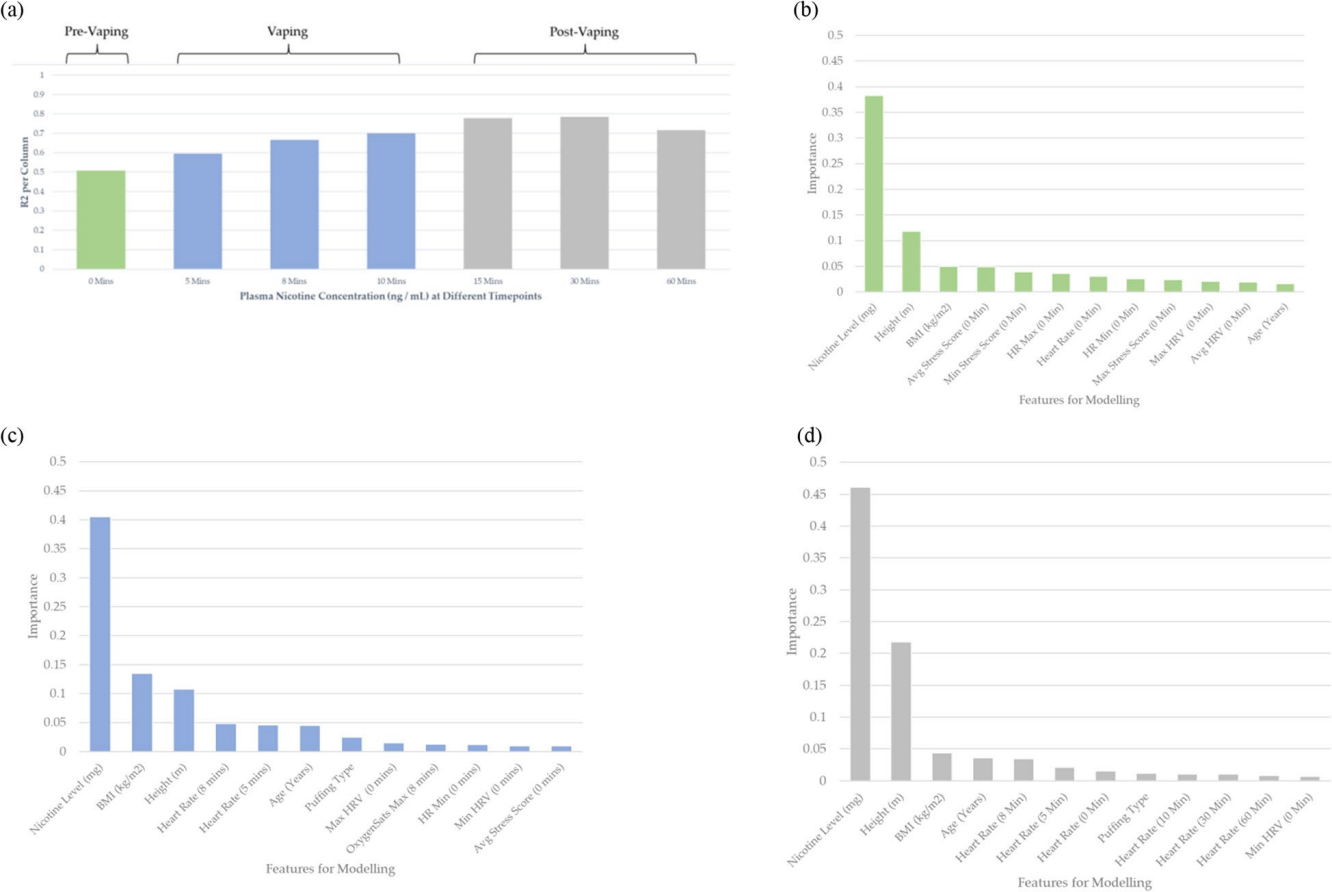
**a** Coefficient of determination (*R*^2^) of different plasma nicotine concentration time points. The bar plot colours indicate the three key experimental regimes; pre-vaping, vaping, and post-vaping. Importance of the descriptors used for modelling for the three different vaping regimes: **b** pre-vaping; **c** vaping; **d** post-vaping

To examine in more detail the accuracy obtainable using all the available data, a final model was trained using information from every participant. The prediction accuracy of the model on validation data increased from *R*^2^ = 0.526 (Phase 1) to *R*^2^ = 0.700 by the end of Phase 2 (Supplementary Figure S6). The change in root-mean-square error for each endpoint over the three models is also given in Supplementary Figure S8. In addition, the model showed better prediction of plasma nicotine during and after vaping than before (Fig. 9a). Note, ideally all participants’ plasma nicotine levels should be zero at the beginning of the study, which would result in 100% predictive accuracy and an R^2^ = 1. Therefore, since R^2^ was < 1 at 0 min (R2 = 0.5), then there was either 1) insufficient abstinence time prior to the study or 2) some participants using nicotine-containing products during the 12-h abstaining window. From Fig. 9b-d, the product nicotine concentration is found to be the most important predictor within all three puffing regimes, this is expected as nicotine is known to have a strong impact on physiological measurements.

Interestingly, descriptors that contributed most to the prediction model varied depending on the stage of the study (pre-vaping, vaping, and post-vaping) (Fig. 9b–d). Height and age become stronger predictors within the later time periods, which might have some link with a participant’s metabolism. BMI is a particularly useful predictor within the during puffing regime, which is not an output previously investigated.

HR, HRV, and PS are all found to be important predictors for plasma nicotine level within all puffing regimes (Fig. 9a-d). These results corroborate the observations found in Fig. 5, whereby these indicators demonstrated a strong qualitative correlation with average nicotine levels.

## Limitations

This study marks a preliminary effort in modelling nicotine pharmacokinetics with less invasive methods, but is still exploratory in nature. The key limitations include:

### Exploratory nature

 The study is at an exploratory stage, highlighting the preliminary nature of the model and the need for further research, validation, and refinement. The use of R2 for model fit, while standard, may not fully capture all aspects of predictive accuracy, particularly across different scales and contexts.

### Participant data constraints

 The study's participant range was limited, primarily based on a single individual, underscoring the need for a broader, more diverse participant sample in future research to enhance applicability.

### Product-specific application

 The model's current applicability is limited to a specific vapour product (ePen3). To ensure generalisability, future studies should test a variety of nicotine products.

### Study design considerations

 Adjustments in study design, such as caffeine incorporation and extended abstinence periods, may have influenced outcomes and should be carefully considered in future research designs.

### Need for clinical validation

 Comprehensive clinical validation, comparing model predictions with actual plasma nicotine levels, is critical for evaluating both accuracy and practical utility.

### Device and participant selection factors

 Further clarification is needed regarding the impact of device modifications and the rationale behind participant selection and sensor usage.

### Model evaluation metrics

 Although R2 was utilised for hyperparameter optimisation, the study does not delve into detailed error prediction for each endpoint. Future research might benefit from incorporating RMSE or other metrics to gain a more detailed understanding of model performance.

In essence, while the study provides a foundational step for computational nicotine pharmacokinetics modelling, extensive work is required to enhance its validity, reliability, and generalisability.

## Conclusions

This study has highlighted important factors that influence the effect of nicotine on the human body, such as BMI and age, as well as key physiological measures that correlate with plasma nicotine levels following vapour product use, such as heart rate, heart rate variability, and physiological stress. The Machine Learning (ML) model also identified these descriptors to be important predictors for modelling nicotine concentration within the blood. These findings were further supported by minimal physiological changes in participants within a control group using non-nicotine vapour products.

Historic nicotine Pharmacokinetic (PK) data was used to train a ML model for the prediction of nicotine plasma levels. Incorporating additional physiological measures from the Phase 1 study led to a reasonable prediction accuracy in cross-validation (*R*^2^ = 0.526) with an improvement of 18% over the PK data-only model (*R*^2^ = 0.446). The inclusion of more diverse data from Phase 2 (including longer baseline measurements, different strength nicotine products, control data from non-nicotine products, and participant information from questionnaires) further improved the prediction accuracy by 57% (*R*^2^ = 0.700) over the preliminary PK-data only model. Predictive performance was higher during the post-puffing period than during product use.

Overall, findings suggest that physiological measures have the potential to act as a ‘digital twin’ for nicotine PK data, facilitating the collection of real-world data, enabling a pre-screening process of physiological parameters among participants prior to clinical studies, and potentially reducing the number of clinical PK studies needed. However, validation of the model in a clinical setting is needed in future studies. In addition, gathering more data from a larger number of participants will reduce the uncertainty in the predictions and develop a better and more generalised model.

### Recommendations

Clinical PK Testing and AI Model Validation: We recommend designing and executing typical pharmacokinetic (PK) testing in a clinical environment. This would involve monitoring real-time physiological parameters while conducting blood draws. The collected physiological data can then be input into the AI model to predict the PK curve. Comparing this predicted PK curve with the actual PK curve, derived from plasma nicotine concentrations obtained from blood draws, will provide a robust test of the AI model’s performance on unseen data.

Comprehensive Model Evaluation Metrics: In addition to using R2, we suggest employing root mean square error (RMSE) or other relevant metrics. This will allow for a more comprehensive evaluation of the model’s predictive errors and overall accuracy, enhancing our understanding of the model's practical applicability and robustness.

### Future research and implications

Preliminary Findings and Model Development: Our findings, while promising, represent an initial phase in the development of a machine learning (ML) model for predicting plasma nicotine levels from physiological parameters. The full realisation of this research holds substantial potential implications. A validated model could significantly streamline the evaluation process for new nicotine and tobacco products, reducing reliance on invasive PK studies and expediting the assessment of product safety and efficacy. After further validation there would be no intention to replace clinical measurements or clinical studies, this exploratory capability would be used only as a pre-screen tool to inform clinical study designs.

Next Steps in Research:

- Clinical Validation: The immediate next step is to validate the model in a clinical setting. This will involve comparing the model's predictions against actual observed nicotine plasma levels under controlled conditions.
- Data Expansion for Accuracy and Generalisability: To refine the model's accuracy and extend its applicability, we plan to collect data from a broader and more diverse participant group. This will include gathering longer baseline measurements, incorporating a variety of nicotine strengths, and including control data from non-nicotine products.
- Technological Integration: Integrating the model with connected e-cigarette devices or wearable health monitors could offer real-time insights into nicotine uptake. This integration would facilitate immediate adjustments in nicotine product usage, tailoring it to individual needs.

Implications of Research:

- Enhanced Screening for Nicotine Products: Once validated, the model could act as an effective tool for preliminary screening of nicotine products, identifying potential risks and efficacies prior to more extensive clinical trials.
- Personalised Health Insights: For individual users, the model could provide personalised feedback on nicotine consumption, potentially aiding in harm reduction or cessation efforts.
- Broader Applications in Pharmacology and Substance Use Research: The methodologies and insights gleaned from this study could be applied to other areas of pharmacological research, potentially revolutionising our approach to studying substance absorption and its effects on the body.

Commitment to Research Progress: We are dedicated to advancing this field of research. The successful development and validation of this ML model are expected to offer considerable benefits to public health and the scientific community, bridging the gap between statistical modelling and clinical application.

### Supplementary Information

Supplementary Material 1.

## Data Availability

The authors declare that the supporting data for the findings of this study are available within the paper. Data sets generated during the study are available from the corresponding author on reasonable request.
