# Supplementary material for: Modelling the nicotine pharmacokinetic profile for e-cigarettes using real time monitoring of consumers’ physiological measurements and mouth level exposure
Source: BioData Min. 2024 Jul 17;17:24. doi: 10.1186/s13040-024-00375-z (PMC11253374; doi:10.1186/s13040-024-00375-z)
Supplement: Supplementary file 1 — Supplementary Material 1. [file 13040_2024_375_MOESM1_ESM.docx]

**Supplementary Tables**

**Supplementary Table S1.** Sensory activation and frequency information relating to ART.

| **Sensor** | **Activation** | **Frequency of readings** | **End** |
| --- | --- | --- | --- |
| Flow | On button press | 25 times per second | Max 6 s after initial draw |
| Temperature | On button press | Single reading | – |
| Humidity | On button press | Single reading | – |
| Pressure | On button press | Single reading | – |
| Accelerometer | On button press | Twice per second | When no flow is detected |

**Supplementary Table S2**. E-liquid formulations.

| **E-liquid** | **Nicotine (mg/ml)** | **Flavour 1 (%)** | **Flavour 2 (%)** | **Flavour 3 (%)** | **PG (%)** | **VG (%)** | **Nicotine (%)** | **Water (%)** |
| --- | --- | --- | --- | --- | --- | --- | --- | --- |
| **Phase 1**  Wild Berries | 18 | 20 | 0.96 | 0.64 | 32.36 | 34.22 | 1.78 | 10 |
| Dark Cherry | 12 | 15.94 | 10.63 | – | 27.44 | 34.71 | 1.29 | 10 |
| Dark Cherry | 0 | 15.94 | 10.63 | – | 27.44 | 36 | 0 | 10 |
| **Phase 2**  Dark Cherry | 18 | 15.94 | 10.63 | – | 27.44 | 34.218 | 1.782 | 10 |
| Dark Cherry | 12 | 15.94 | 10.63 | – | 27.44 | 34.692 | 1.308 | 10 |
| Dark Cherry | 6 | 15.94 | 10.63 | – | 27.44 | 35.34 | 0.66 | 10 |
| Dark Cherry | 0 | 15.94 | 10.63 | – | 27.44 | 36 | 0 | 10 |
| Mint | 18 | 4.50 | 2.36 | 0.18 | 46.96 | 34.218 | 1.782 | 10 |
| Mint | 0 | 4.50 | 2.36 | 0.18 | 46.96 | 36 | 0 | 10 |
| Blended Tobacco | 18 | 22.28 | N/A | N/A | 31.722 | 34.218 | 1.782 | 10 |
| Blended Tobacco | 6  Abbreviations: PG, propylene glycol; VG, vegetable glycerine. | 22.28 | N/A | N/A | 31.722 | 36 | 0 | 10 |
|  |  |  |  |  |  |  |  |  |

**Supplementary Table S3.** Data collection points in Phases 1 and 2.

| Stage | Phase 1 | Phase 2 |
| --- | --- | --- |
| Pre-vaping | - |  |
| Pre-vaping | - | –50 min |
| Pre-vaping | - | –40 min |
| Pre-vaping | - | –30 min |
| Pre-vaping | - | –20 min |
| Pre-vaping | –10 min | –10 min |
| Pre-vaping | –2–5 min | – |
| Vaping – start | 0 min | 0 min |
| Vaping | 4–5 min | 5 min |
| Vaping | 8 min | 8 min |
| Vaping – end | 10 min | 10 min |
| Post-vaping | 12 min | – |
| Post-vaping | 15 min | – |
| Post-vaping | - | 20 min |
| Post-vaping | 30 min | 30 min |
| Post-vaping | 60 min | – |

**Supplementary Table S4.** Device mass loss in Phase 1.

| **Participant ID** | **Pre weight (mg)** | **Post weight (mg)** | **DML (mg)** |
| --- | --- | --- | --- |
| ART – 12 mg/mL  nic 30 | 13.0608 | 12.9765 | 0.0843 |
| 28 | 12.9899 | 12.8973 | 0.0926 |
| 32 | 13.0504 | 13.0012 | 0.0492 |
| 31 | 13.0527 | 12.6835 | 0.3692 |
| 29 | 13.1469 | 13.0535 | 0.0934 |
| 27 | 13.0205 | 12.6835 | 0.3370 |
| 26 | 13.0916 | 13.0501 | 0.0415 |
| 24 | 12.9841 | 12.8918 | 0.0923 |
| 17 | 13.0844 | 13.0463 | 0.0381 |
| 33 | 13.0151 | 12.9376 | 0.0775 |
| 34 | 13.0276 | 12.8666 | 0.1610 |
| 35 | 13.0702 | 12.8882 | 0.1820 |
| 36 | 13.0740 | 13.0023 | 0.0717 |
| ART – 0 mg/mL  32 | 13.1313 | 13.0454 | 0.0859 |
| 29 | 13.0147 | 12.8592 | 0.1555 |
| 27 | 12.9803 | 12.9113 | 0.069 |
| 17 | 13.0200 | 13.0173 | 0.0027 |

**Supplementary Table S5**. Device mass loss in Phase 2.

### Participant ID Pre weight (mg) Post weight (mg) DML (mg)

18 mg/mL nic

1. 13.0404 12.9927 0.0477
2. 13.0962 12.8959 0.2003

24 13.1161 12.9952 0.1209

1. 13.0468 12.9941 0.0527
2. 13.0941 13.0168 0.0773
3. 13.0326 12.9287 0.1039
4. 13.1234 12.9513 0.1721
5. 13.0267 12.8089 0.2178
6. 13.0395 12.9848 0.0547
7. 13.1313 13.0540 0.0773
8. 13.1479 12.9953 0.1526
9. 13.1435 12.993 0.1505
10. 13.1819 13.1035 0.0784
11. 13.1083 13.0042 0.1041
12. 13.1292 12.901 0.2282
13. 13.0658 12.9442 0.1216

12 mg/mL nic

22 13.1142 13.0228 0.0914

6 mg/mL nic

23 13.0170 12.9090 0.1080

0 mg/mL nic

1. 13.1228 13.0521 0.0707
2. 13.114 12.9527 0.1613
3. 13.1536 12.9889 0.1647
4. 13.0177 12.8937 0.1240
5. 13.1524 12.9441 0.2083
6. 13.0574 12.9591 0.0983
7. 13.2149 12.9993 0.2156
8. 13.1494 13.0153 0.1341
9. 13.0165 12.8208 0.1957
10. 13.1222 12.8647 0.2575
11. 13.0852 13.0169 0.0683
12. 13.0989 12.9934 0.1055
13. 13.1368 12.9598 0.1770

35* 13.1156 12.8734 0.2422

1. 13.0140 12.9122 0.1018
2. 13.1234 12.9266 0.1968
3. 13.0580 12.9809 0.0771

39* 13.0799 12.8801 0.1998

40 13.1234 12.9266 0.1968

*Due to noise on first recording, participants 35 and 39 repeated the session and the second data only are reported here

### Supplementary Figures


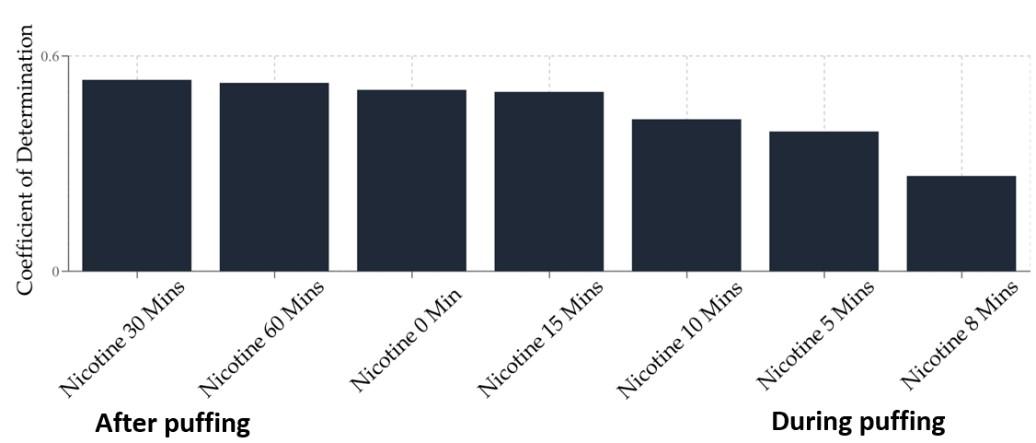


**Supplementary Figure S1.** Accuracy of the preliminary model based on historic nicotine PK data to predict nicotine plasma levels at different experimental time points.


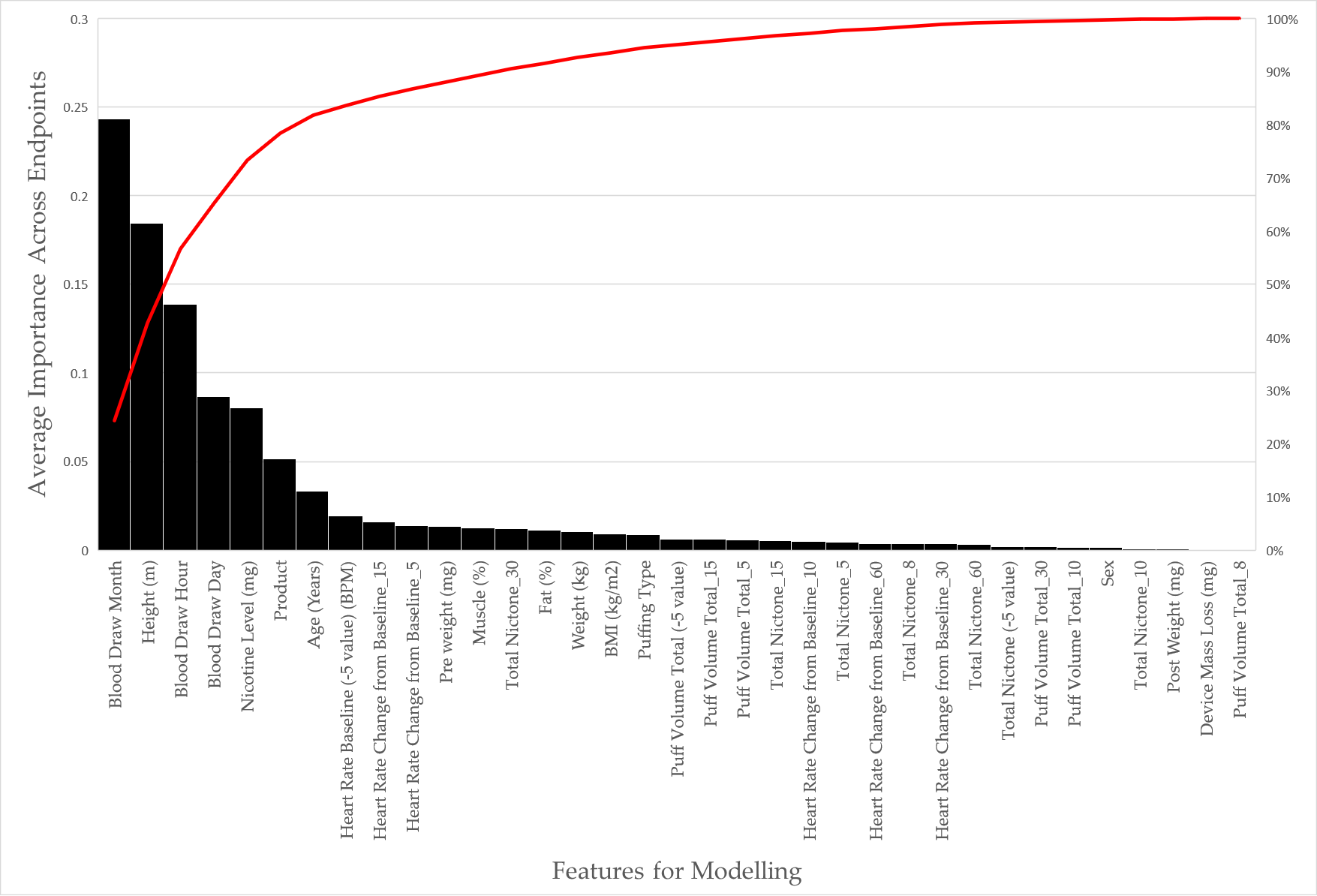


**Supplementary Figure S2.** Inputs used by the preliminary model to predict one or more targets.

#### NICOTINE GROUP (Subject ID) NON-NICOTINE GROUP (Subject ID)

|  |  | | | | | | | | | | | | | | | | | | |  |  | | | | | | | | | | | | | | | | | | |
| --- | --- | --- | --- | --- | --- | --- | --- | --- | --- | --- | --- | --- | --- | --- | --- | --- | --- | --- | --- | --- | --- | --- | --- | --- | --- | --- | --- | --- | --- | --- | --- | --- | --- | --- | --- | --- | --- | --- | --- |
| **Time from First Puff (min)** | **P17** | **P18** | **P22** | **P23** | **P24** | **P26** | **P27** | **P28** | **P29** | **P31** | **P32** | **P33** | **P34** | **P35** | **P36** | **P37** | **P38** | **P39** | **P40** |  | **P17** | **P18** | **P22** | **P23** | **P24** | **P26** | **P27** | **P28** | **P29** | **P31** | **P32** | **P33** | **P34** | **P35** | **P36** | **P37** | **P38** | **P39** | **P40** |
| 1 | 3% | 3% | 1% | 2% | 3% | 2% | -4% | 7% | 5% | 2% | 0% | -5% | 14% | 14% | 4% | 4% |  | 9% | 9% |  | 4% | 4% | 2% | 27% | 1% | 2% | 1% | 4% | 9% |  | -5% | -2% | -1% | -3% | -1% | 3% | -2% | 5% | 2% |
| 2 | 3% | -1% | 4% | 2% | 11% | -2% | -1% | 3% | 8% | 5% | 0% | -4% | 8% | 15% | -4% | 13% |  | 24% | 21% |  | -2% | 0% | 2% | -4% | 0% | -7% | -2% | 0% | 0% |  | -1% | -1% | -1% | 3% | -3% | 5% | -5% | 2% | -2% |
| 3 | 18% | 4% | 6% | 4% | 18% | -1% | -6% | 6% | 9% | 12% | -1% | -7% | 13% | 7% | -4% | 19% |  | 26% | 9% |  | -3% | 1% | -1% | -6% | 2% | 0% | -5% | 4% | -4% |  | -3% | -2% | 0% | -4% | -2% | 3% | 1% | 4% | 6% |
| 4 | 11% | 10% | 10% | 7% | 17% | 2% | -3% | 6% | 9% | 12% | 2% | -1% | 18% | 25% | 4% | 22% |  | 22% | 13% |  | 1% | -2% | 2% | -4% | 4% | -3% | -2% | 2% | 0% |  | -4% | -2% | -4% | 1% | 0% | -7% | -8% | 5% | 5% |
| 5 | 12% | 7% | 8% | 11% | 17% | 1% | 0% | 7% | 5% | 9% | 2% | -1% | 21% | 24% | 1% | 28% |  | 24% | 16% |  | -2% | -3% | 1% | -4% | 5% | -4% | -5% | -4% | -3% |  | -3% | 3% | -5% | 3% | -4% | 0% | -6% | 4% | 6% |
| 6 | 15% | 10% | 14% | 9% | 18% | -1% | -1% | 13% | 8% | 13% | 4% | 0% | 24% | 30% | 5% | 32% |  | 26% | 20% |  | 2% | -2% | -1% | -1% | -1% | -2% | -1% | 5% | 4% |  | -3% | 2% | -7% | 0% | 1% | 2% | -1% | 3% | 8% |
| 7 | 14% | 10% | 19% | 10% | 23% | 2% | -1% | 2% | 5% | 11% | -3% | -3% | 21% | 24% | 4% | 36% |  | 31% | 23% |  | -2% | -2% | -2% | 10% | 2% | -6% | -4% | 1% | -3% |  | -5% | 3% | -6% | -3% | 3% | -3% | -8% | 8% | 2% |
| 8 | 13% | 10% | 22% | 11% | 17% | 5% | 0% | 17% | 9% | 14% | -3% | -4% | 19% | 28% | 3% | 32% |  | 29% | 19% |  | 7% | -2% | 1% | -5% | 1% | -10% | -4% | -1% | -6% |  | 0% | -6% | -3% | 2% | -2% | 3% | 0% | 8% | 10% |
| 9 | 13% | 12% | 20% | 12% | 17% | 3% | 4% | 21% | 7% | 8% | -6% | -6% | 24% | 27% | 9% | 46% |  | 29% | 23% |  | 0% | -4% | 1% | -4% | -1% | 0% | -4% | 2% | -1% |  | -5% | -2% | -6% | -1% | -5% | -2% | -4% | 4% | 10% |
| 10 | 14% | 11% | 17% | 15% | 22% | 3% | 1% | 18% | 8% | 13% | -4% | -4% | 23% | 22% | 9% | 44% |  | 29% | 24% |  | -2% | -5% | 2% | 0% | 6% | 1% | -3% | 0% | -4% |  | -3% | 3% | 0% | 0% | -3% | 1% | -3% | 10% | 10% |
| 11 | 10% | 13% | 19% | 13% | 16% | 1% | 0% | 14% | 2% | 11% | -5% | 3% | 34% | 18% | 5% | 39% |  | 22% | 17% |  | 2% | 3% | 5% | 0% | 2% | -4% | -2% | 2% | -4% |  | -1% | 2% | 0% | -3% | 0% | 10% | -1% | 3% | 5% |
| 12 | 12% | 11% | 18% | 6% | 19% | 1% | -2% | 7% | -1% | 12% | 7% | -4% | 13% | 18% | 7% | 35% |  | 23% | 23% |  | 2% | 0% | 5% | -8% | -6% | 2% | -5% | -7% | -2% |  | -8% | -12% | 0% | 1% | -1% | 0% | -5% | 1% | -5% |
| 13 | 11% | 13% | 19% | 5% | 21% | 10% | -1% | 6% | 6% | 0% | 1% | 0% | 14% | 19% | 11% | 36% |  | 21% | 24% |  | 1% | -1% | 6% | -9% | 0% | 1% | -3% | 5% | -2% |  | -10% | -13% | 0% | 3% | -1% | 5% | -1% | -5% | -7% |
| 14 | 14% | 6% | 16% | 6% | 20% | 0% | 1% | 2% | 2% | 13% | -1% | 5% | 14% | 19% | 4% | 37% |  | 20% | 24% |  | -1% | 1% | 4% | -9% | -5% | 9% | -2% | -7% | -7% |  | -6% | -9% | 0% | 0% | -9% | 3% | -8% | -2% | -3% |
| 15 | 10% | 14% | 17% | 10% | 14% | 1% | 0% | 3% | -3% | 12% | -6% | 4% | 16% | 21% | 5% | 44% |  | 23% | 29% |  | -3% | -1% | 6% | -7% | -5% | -7% | 10% | -4% | -3% |  | -3% | -9% | -5% | 0% | -10% | 3% | -13% | -5% | -1% |
| 16 | 10% | 11% | 17% | 4% | 19% | 1% | -3% | 3% | 5% | 16% | -2% | 10% | 13% | 19% | 4% | 32% |  | 17% | 17% |  | -4% | 3% | 5% | -9% | -5% | -3% | -1% | -2% | -6% |  | -7% | -4% | -6% | 1% | -7% | 5% | -9% | -2% | -11% |
| 17 | 16% | 10% | 14% | 4% | 17% | -6% | -4% | 4% | 1% | -2% | 3% | 2% | 17% | 17% | 5% | 53% |  | 11% | 20% |  | -3% | 8% | 2% | -9% | -3% | -1% | -2% | -6% | -3% |  | -7% | -5% | 0% | 2% | -5% | -1% | 2% | -3% | -8% |
| 18 | 9% | 15% | 14% | 11% | 16% | -4% | -1% | 3% | 0% | 4% | -1% | 4% | 14% | 14% | 8% | 23% |  | 15% | 21% |  | -2% | -1% | 0% | -4% | 2% | 0% | 4% | -3% | -7% |  | -10% | -5% | -5% | -6% | -2% | -3% | 1% | -4% | -11% |
| 19 | 8% | 6% | 17% | 2% | 15% | 1% | -6% | 0% | 2% | -1% | 1% | 0% | 12% | 13% | 8% | 49% |  | 12% | 12% |  | 0% | 0% | 7% | -4% | -4% | 11% | 2% | -2% | -5% |  | -6% | -7% | -6% | -5% | -8% | -12% | -6% | -3% | -10% |
| 20 | 10% | 9% | 10% | 1% | 15% | 4% | -6% | 1% | 3% | -3% | 4% | 1% | 15% | 15% | 8% | 57% |  | 8% | 12% |  | -6% | 1% | 1% | -5% | -9% | 12% | 0% | -1% | -2% |  | -10% | 0% | -7% | 1% | -2% | 10% | -10% | -5% | -5% |
| 21 | 9% | 14% | 12% | 5% | 16% | 7% | -9% | 5% | -1% | -2% | 1% | 4% | 7% | 18% | 6% | 31% |  | 9% | 14% |  | -5% | 8% | -2% | 0% | -3% | 4% | -3% | 2% | -2% |  | -1% | -3% | -8% | -1% | -8% | 0% | -10% | -3% | -16% |
| 22 | 11% | 12% | 10% | 2% | 12% | 8% | -8% | 1% | 1% | 5% | -1% | -8% | 10% | 12% | 9% | 20% |  | 12% | 13% |  | -3% | -3% | 2% | -9% | -3% | 4% | -6% | -7% | -7% |  | -13% | -9% | -2% | -6% | -6% | -3% | -12% | -4% | -13% |
| 23 | 10% | 12% | 13% | 0% | 6% | 2% | -7% | -2% | 5% | 11% | 0% | -9% | 11% | 12% | 8% | 31% |  | 7% | 18% |  | -2% | 1% | 3% | -8% | -4% | 6% | -5% | -4% | -3% |  | -9% | -9% | -9% | -3% | -8% | -2% | -3% | -4% | -11% |
| 24 | 11% | 8% | 14% | 7% | 7% | -8% | -8% | 2% | 5% | 6% | 0% | -9% | 21% | 13% | 6% | 19% |  | 6% | 11% |  | -4% | -1% | -1% | -8% | -4% | 0% | -6% | -4% | -5% |  | 1% | -5% | -7% | -7% | -6% | 8% | 6% | -2% | -14% |
| 25 | 8% | 8% | 11% | 2% | 3% | -5% | -8% | 4% | 4% | 6% | -4% | -10% | 10% | 10% | 4% | 31% |  | 11% | 16% |  | 1% | 2% | 0% | -6% | -4% | 9% | -5% | -2% | 3% |  | -14% | -9% | -3% | 3% | -10% | 2% | -10% | -4% | -6% |
| 26 | 9% | 6% | 10% | 1% | 2% | -4% | -9% | 1% | -2% | 4% | -3% | -7% | 5% | 13% | 7% | 33% |  | 8% | 19% |  | 1% | 0% | 1% | 2% | -6% | -2% | -16% | 4% | -5% |  | -9% | -2% | -9% | 1% | -6% | -7% | -11% | -3% | -11% |
| 27 | 11% | 12% | 9% | 4% | 5% | -4% | -6% | 3% | -1% | 3% | -4% | -6% | 7% | 13% | 1% | 0% |  | 7% | 16% |  | -4% | 1% | 1% | -2% | -9% | 3% | -7% | -5% | -5% |  | -8% | -3% | -7% | 1% | -6% | -3% | -8% | -5% | -7% |
| 28 | 10% | 9% | 11% | 8% | 6% | -1% | -9% | -7% |  | 1% | -10% | -2% | 8% | 13% | 1% | 14% |  | 4% | 14% |  | -4% | 0% | 0% |  | -10% | 4% | -15% | -7% | -9% |  | -11% | -4% | -10% | -8% | -9% | 2% | 1% | -4% | -7% |
| 29 | 4% | 9% | 8% | 2% | 6% | 7% | -7% | 11% |  | 6% | -8% | -8% | 5% | 11% | 3% | 35% |  | 12% | 11% |  | -1% | 0% | 3% | -15% | -7% | -8% | -16% | -9% | -7% |  | -9% | 4% | -3% | 0% | -10% | -6% | -1% | -2% | -12% |
| 30 | 6% | 8% |  | 12% | 10% | 1% | -4% | 2% |  | -7% | -3% | 4% | 3% | 14% | 7% | 15% |  | 14% | 9% |  | -1% | -1% | -2% | -10% | -2% | 0% | -16% | -7% | -5% |  | -6% | -6% | 0% | -3% | -6% | -7% | -10% | -2% | -7% |

**Supplementary Figure S3.** Percentage HR change relative to baseline value per participant (P17 to P40). Shading goes from Green, indicates a strong increase in HR relative to baseline, through Amber, slight decrease in HR relative to baseline to Red, a strong increase in HR relative to baseline. The left-hand and right-hand tables represent nicotine and non-nicotine groups, respectively.

-5.00

-4.00

-3.00

-2.00

-1.00

0.00

1.00

2.00

3.00

4.00

5.00

0

5

10

15

20

25

30

35

Delta BR (rpm)

Time from first puff (min)

Mean Delta BR (rpm) from Time Zero Baseline

Nicotine Group Average

Non-Nicotine Group Average

**Supplementary Figure S4.** Mean absolute change in breathing rate in the nicotine and non-nicotine groups (control) relative to baseline.

-10

%

%

-5

0

%

5

%

10

%

15

%

%

20

25

%

30

%

%

35

%

40

0

5

10

15

20

25

30

Delta GSR (%)

Time from first puff (min)

Mean Delta GSR (%): Individual Nicotine

-

Non

-

Nicotine

**Supplementary Figure S5.** Mean percentage change in GSR.


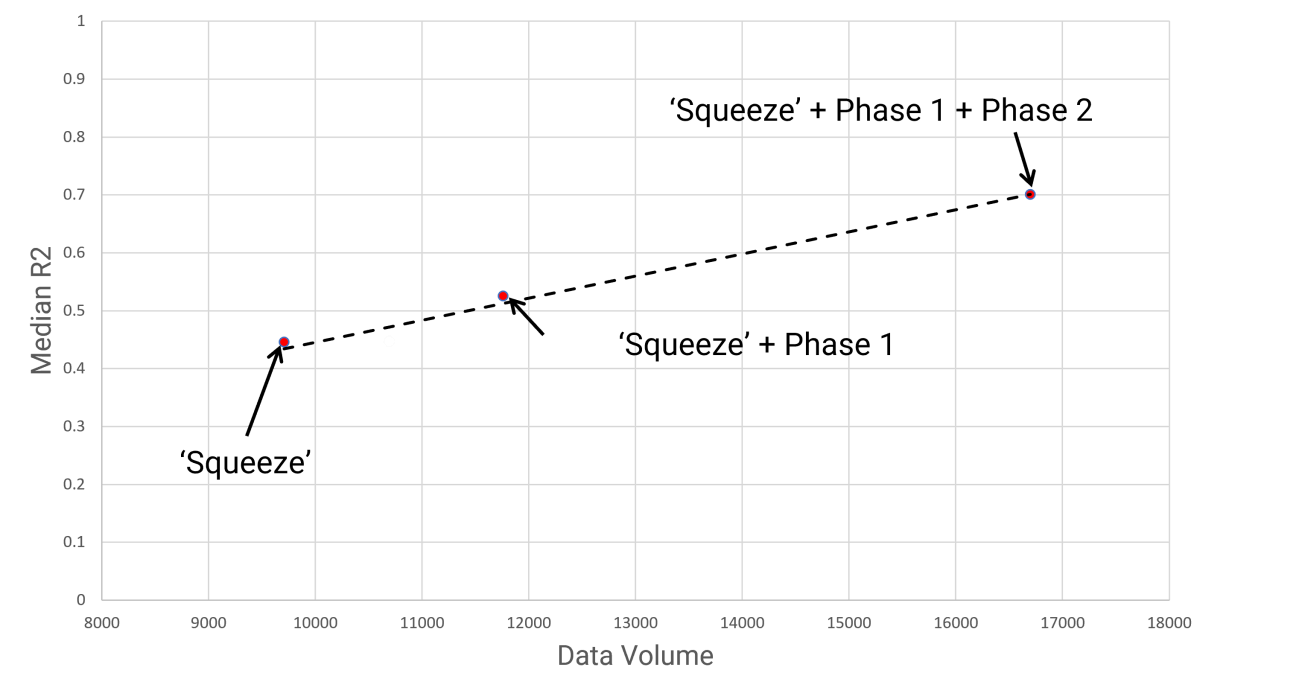


**Supplementary Figure S6.** Median R2 change as data ingestion into model increases.


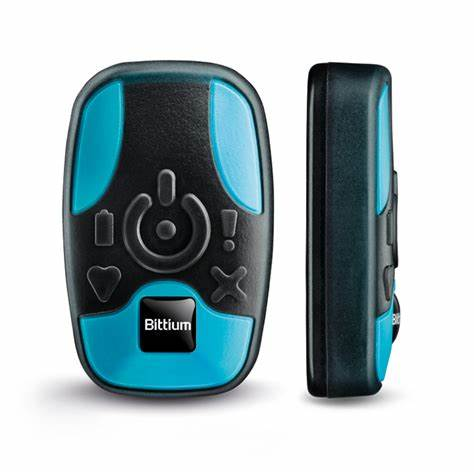


**Supplementary Figure S7.** Bittium Faros 180 Monitor

Supplementary Questionnaire **SQ1** Before Testing:

Have you exercised [Free Text Box]

Have you consumed any Caffeine today [Free Text Box]

How you has your day been [Free Text Box] After Testing:

Have you exercised [Free Text Box]

Have you consumed any Caffeine today [Free Text Box]

How you has your day been [Free Text Box]

Age: [Free Text Box]

Biological Sex: [Free Text Box]

Height: [Free Text Box]

Weight: [Free Text Box]

Average daily Nicotine Intake: [Free Text Box]

*[All questions above will require a tick box option for ‘do not want to share this data’ to ensure participants aren’t pressured to fill in their data]*

**Supplementary Figure S8.** RMSE for different endpoints and data/model metrics for the three models developed

|  | | Preliminary Model | Phase 1 Model | Phase 2 Model |
| --- | --- | --- | --- | --- |
| RMSE | LN Nicotine Baseline (-5 Nic value) (log ng/mL) | 1.969 | 0.384 | 0.405 |
|  | LN Nicotine Change from Baseline_5 (log ng/mL) | 0.549 | 0.533 | 0.523 |
|  | LN Nicotine Change from Baseline_8 (log ng/mL) | 0.467 | 0.46 | 0.483 |
|  | LN Nicotine Change from Baseline_10 (log ng/mL) | 0.462 | 0.455 | 0.484 |
|  | LN Nicotine Change from Baseline_15 (log ng/mL) | 0.383 | 0.346 | 0.4 |
|  | LN Nicotine Change from Baseline_30 (log ng/mL) | 0.328 | 0.327 | 0.362 |
|  | LN Nicotine Change from Baseline_60 (log ng/mL) | 0.35 | 0.332 | 0.362 |
|  | T_Max (min) |  |  | 5.177 |
|  | C_Max (log ng/mL) |  |  | 0.454 |
|  | | | | |
| Data / Model Metrics | Rows | 401 | 410 | 455 |
|  | Columns | 24 | 100 | 162 |
|  | Data Quantity | 9575 | 10495 | 14723 |
|  | Median R2 | 0.446 | 0.526 | 0.7 |
